# Expansion of a urethritis-associated *Neisseria meningitidis* clade in the United States with concurrent acquisition of *N. gonorrhoeae* alleles

**DOI:** 10.1186/s12864-018-4560-x

**Published:** 2018-03-02

**Authors:** Adam C. Retchless, Cécilia B. Kretz, How-Yi Chang, Jose A. Bazan, A. Jeanine Abrams, Abigail Norris Turner, Laurel T. Jenkins, David L. Trees, Yih-Ling Tzeng, David S. Stephens, Jessica R. MacNeil, Xin Wang

**Affiliations:** 1 Division of Bacterial Diseases, National Center for Immunization and Respiratory Diseases, Centers for Disease Control and Prevention, Atlanta, GA USA; 2 Division of Infectious Diseases, Department of Internal Medicine, Ohio State University College of Medicine, Columbus, OH USA; 3 Sexual Health Clinic, Columbus Public Health, Columbus, OH USA; 4 Division of STD Prevention, National Center for HIV/AIDS, Viral Hepatitis, STD and TB Prevention, Centers for Disease Control and Prevention, Atlanta, GA USA; 5 Division of Infectious Diseases, Department of Medicine, Emory University School of Medicine, Atlanta, GA USA; 6 Department of Microbiology and Immunology, Emory University School of Medicine, Atlanta, GA USA; 7 Present address: Division of Scientific Education and Professional Development, Center for Surveillance, Epidemiology and Laboratory Services, Centers for Disease Control and Prevention, Atlanta, GA USA

**Keywords:** *Neisseria meningitidis*, Genital disease, Gene transfer, Speciation, *Neisseria gonorrhoeae*

## Abstract

**Background:**

Increased reports of *Neisseria meningitidis* urethritis in multiple U.S. cities during 2015 have been attributed to the emergence of a novel clade of nongroupable *N. meningitidis* within the ST-11 clonal complex, the “U.S. NmNG urethritis clade”. Genetic recombination with *N. gonorrhoeae* has been proposed to enable efficient sexual transmission by this clade. To understand the evolutionary origin and diversification of the U.S. NmNG urethritis clade, whole-genome phylogenetic analysis was performed to identify its members among the *N. meningitidis* strain collection from the Centers for Disease Control and Prevention, including 209 urogenital and rectal *N. meningitidis* isolates submitted by U.S. public health departments in eleven states starting in 2015.

**Results:**

The earliest representatives of the U.S. NmNG urethritis clade were identified from cases of invasive disease that occurred in 2013. Among 209 urogenital and rectal isolates submitted from January 2015 to September 2016, the clade accounted for 189/198 male urogenital isolates, 3/4 female urogenital isolates, and 1/7 rectal isolates. In total, members of the clade were isolated in thirteen states between 2013 and 2016, which evolved from a common ancestor that likely existed during 2011. The ancestor contained *N. gonorrhoeae*-like alleles in three regions of its genome, two of which may facilitate nitrite-dependent anaerobic growth during colonization of urogenital sites. Additional gonococcal-like alleles were acquired as the clade diversified. Notably, one isolate contained a sequence associated with azithromycin resistance in *N. gonorrhoeae*, but no other gonococcal antimicrobial resistance determinants were detected.

**Conclusions:**

Interspecies genetic recombination contributed to the early evolution and subsequent diversification of the U.S. NmNG urethritis clade. Ongoing acquisition of *N. gonorrhoeae* alleles by the U.S. NmNG urethritis clade may facilitate the expansion of its ecological niche while also increasing the frequency with which it causes urethritis.

**Electronic supplementary material:**

The online version of this article (10.1186/s12864-018-4560-x) contains supplementary material, which is available to authorized users.

## Background

*Neisseria meningitidis* is primarily recognized as a cause of invasive, life-threatening infections such as meningitis and septicemia [1]. However, *N. meningitidis* is also an occasional cause of other infections, including urethritis [2–6]. Several isolates from cases of meningococcal urethritis in France and Germany were recently found to be closely related to isolates from cases of invasive meningococcal disease among men who have sex with men (MSM) in these countries [7], reviving a hypothesis that urethral colonization may contribute to invasive disease among MSM [8–10]. Recently, notable increases in the number of *N. meningitidis* urethritis cases were reported from two U.S. sexually transmitted disease clinics in Columbus, Ohio and Oakland County, Michigan [11]. For instance, in Columbus, *N. meningitidis* was isolated from 75 urethritis cases between January and November of 2015, equal to a quarter of the *N. gonorrhoeae* isolated during that period [12].

These *N. meningitidis* isolates formed a novel, urethritis-associated clade [12], which also includes recently described urethritis isolates from Georgia and Indiana [8, 13]. This clade is part of the hypervirulent lineage 11 of *N. meningitidis*, corresponding to the ST-11 clonal complex (CC11) [14]. This is the same lineage associated with several outbreaks of invasive meningococcal disease among MSM [7, 15–18]. Unlike the isolates from meningitis outbreaks among MSM, which were assigned to *N. meningitidis* serogroup C (NmC) due to the structure and immunogenic properties of their bacterial capsules, the urethritis-associated isolates identified by Bazan et al. [12] are nongroupable *N. meningitidis* (NmNG) because of a multi-gene deletion at the capsule synthesis locus [8]. The bacterial capsule influences traits such as resistance to serum bactericidal activity and adhesion to epithelial cells [19]. The capsule is notably absent from urogenital pathogen *N. gonorrhoeae*, which is the most closely related species to *N. meningitidis* and a common cause of urethritis [20].

The clade identified by Bazan et al. [12] (hereafter, the “U.S. NmNG urethritis clade”) is also similar to *N. gonorrhoeae* in that the isolates are capable of nitrite dependent anaerobic growth [8]. This phenotype corresponds to the presence of gonococcal-like *aniA* and *norB* genes, which catalyze the conversion of nitrite to nitrous oxide, by way of nitric oxide. Gene flow between *N. meningitidis* and *N. gonorrhoeae* has rarely been detected [20, 21], so acquisition of *aniA* and *norB* from *N. gonorrhoeae* through homologous recombination raises the prospect that other loci may also undergo horizontal gene transfer between these two exclusively human-colonizing bacteria. Transfer of antimicrobial resistance determinants are of particular concern, since antimicrobial resistance is common in *N. gonorrhoeae* [22, 23] but rare in *N. meningitidis* in the United States [24]. Illustrating this risk, a *N. meningitidis* strain with a *N. gonorrhoeae* penicillin resistance-associated *penA* allele was recently reported from an invasive meningococcal disease case involving a patient on long-term complement inhibitor therapy and daily penicillin chemoprophylaxis [25].

The Gonococcal Isolate Surveillance Project (GISP) is a network of 27 sexually transmitted disease clinics that routinely collect isolates from men with signs or symptoms of urethritis in order to monitor trends in antimicrobial susceptibility in *N. gonorrhoeae* [11, 23]. Urethral infections involving the U.S. NmNG urethritis clade were initially identified at two GISP sentinel sites by the presence of discordant laboratory test results: observation of gram-negative intracellular diplococci (GNID) in urethral secretions and growth of oxidase-positive bacterial colonies on selective media (Modified Thayer-Martin), but negative results for *N. gonorrhoeae* nucleic acid amplification testing (NAAT) of urine [11]. The Centers for Disease Control and Prevention (CDC) subsequently requested that public health laboratories submit cultures of suspected *N. meningitidis* isolated from body sites typically associated with *N. gonorrhoeae* infection, including the rectum and urogenital tract.

The genomes of these *N. meningitidis* isolates were sequenced to assess whether additional clades of *N. meningitidis* are associated with urethritis and to characterize the geographic distribution of the previously identified U.S. NmNG urethritis clade. Further analyses were conducted to identify cases of invasive disease involving the U.S. NmNG urethritis clade, the evolutionary relationship between *N. meningitidis* urethritis isolates and invasive disease isolates from MSM, and the genetic changes that corresponded to the emergence of this novel urethritis pathogen.

## Results

### The U.S. NmNG urethritis clade includes invasive disease isolates

Twelve isolates in the CDC *N. meningitidis* strain collection were identified as belonging to the U.S. NmNG urethritis clade, not including isolates that were submitted in response to the previously described clusters of *N. meningitidis* urethritis (i.e. urogenital and rectal isolates collected after January 1, 2015, described below). All 12 isolates were nongroupable. Of these 12 isolates, five were from patients with invasive disease that occurred between 2013 and 2016. Two of the five were cultured from cerebrospinal fluid (CSF) of patients with meningitis and the remaining three isolates were cultured from the blood of patients. Four of the five patients were males (two of whom had sex with men) and one was female.

The remaining seven U.S. NmNG urethritis clade isolates identified within the CDC strain collection were not from invasive disease. One male urogenital isolate was recovered in New York in 2013, and another was recovered in Michigan in 2014. Three isolates were collected from the mouth and throat, where one originated from an oral swab provided by a sexual partner of a urethritis patient previously known to be infected by an isolate from the U.S. NmNG urethritis clade. An additional two isolates were collected from eye swabs.

### Most recent urethritis isolates belong to the U.S. NmNG urethritis clade

As a result of the CDC request to state and local public health departments (see Methods), a total of 209 *N. meningitidis* isolates collected from urogenital and rectal sites between January 1, 2015 and September 30, 2016 were obtained, sequenced, and analyzed (Table 1, Additional file 1). This included 75 previously described urethral isolates from Columbus, Ohio [12]. Isolates collected from the male urogenital system (*n* = 198) were recovered in 11 states. The remaining isolates (*n* = 11) were from the rectum or female urogenital system, which also were recovered in three of those states. The vast majority of isolates (*n* = 195) belonged to CC11, with the remaining 14 isolates (six rectal and eight urogenital) belonging to 14 different clonal complexes (three of which are not formally designated).

**Table 1:** Urogenital and rectal *N. meningitidis* isolates submitted to the CDC: January 2015–September 2016

		Year		Serogroup		
Body site	Total	2015	2016	NG	B	Y
Male urogenital system	198	124	74	196	1	1
Rectum	7	6	1	3	4	0
Female urogenital system	4	2	2	4	0	0
Total	209	132	77	203	5	1

NG = nongroupable

**Fig. 1:**
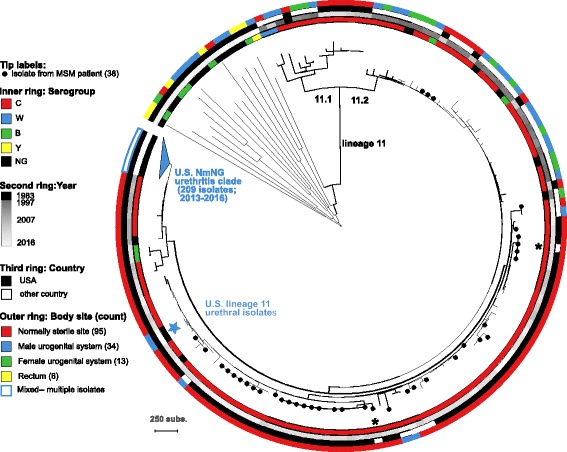
SNP parsimony phylogeny of 353 *N. meningitidis* isolates. Isolates are marked to indicate the serogroup (inner ring), year of isolation (second ring), whether collected in the USA (third ring), the body site from which they were isolated (outer ring), and whether the patient was a MSM (circles on branch tips). Counts in the key refer to isolates outside of the U.S. NmNG urethritis clade. The 209 isolates of the U.S. NmNG urethritis clade are collapsed, with details presented in Fig. 2. Additional labels identify the lineage 11 branch, the branches for sublineages 11.1 and 11.2, two European isolates from MSM that are closely related to U.S. isolates from MSM (asterisks), and two closely related NmNG urethritis isolates within sublineage 11.2 (blue star). The scale bar represents 250 substitutions within the 8607 core SNP alignment

A SNP-based phylogeny of these 209 isolates was inferred to identify clades associated with urogenital or rectal colonization, as well as their relationship to 144 isolates from urogenital and invasive meningococcal disease cases, among both MSM and non-MSM patients. (Fig. 1; collection described in methods and Additional files 1 and 2). The U.S. NmNG urethritis clade is in sublineage 11.2, and included 193 of the 209 isolates submitted for this study (189 male urogenital, three female urogenital, one rectal). Two additional NmNG urethral isolates submitted for this study were also in sublineage 11.2, and were most closely related to each other (Fig. 1, blue star, 99.9% bootstrap support). The remaining 14 urogenital and rectal isolates submitted for this study were distantly related from each other and the lineage 11 isolates.

Among the 144 isolates compared to the U.S. urogenital and rectal isolates, the closest relatives were among the 44 CC11 isolates from invasive case-patients who are not known to be MSM. The 33 CC11 urogenital isolates from outside the United States (Fig. 1, white in third ring, blue in outer ring) were not the closest relatives of any urogenital or rectal isolates collected in the United States (black in third ring, blue in outer ring); nor were the 38 CC11 isolates from invasive cases among MSM (Fig. 1, circle on branch tip, red in outer ring).

Isolates from invasive cases among MSM in the United States were phylogenetically dispersed among isolates from invasive cases among non-MSM patients, except for previously described outbreaks [16–18, 26]. However, two European isolates from invasive cases among MSM were similar to two different groups of U.S. isolates collected during outbreaks of invasive disease among MSM (Fig. 1, asterisks; LNP27256 from France and M11 240,294 from the U.K.).

Besides the 193 isolates submitted for this study and 12 isolates identified among the genomes in the CDC strain collection (as described above), the U.S. NmNG urethritis clade also included four urethral isolates from Indiana with publicly available genome assemblies.

The remaining isolates in this analysis were three invasive NmNG isolates belonging to CC11, CC192, and CC41/44, two sublineage 11.1 genomes included as phylogenetic references (FAM18 and the serogroup W Hajj-related outbreak isolate M07149), and eight invasive disease isolates identified as the closest relatives of the U.S. NmNG urethritis clade in the CDC strain collection. They were collected from invasive cases in 2007 and 2008; seven were NmC and one was NmNG.

### The U.S. NmNG urethritis clade has recently spread to multiple states

**Fig. 2:**
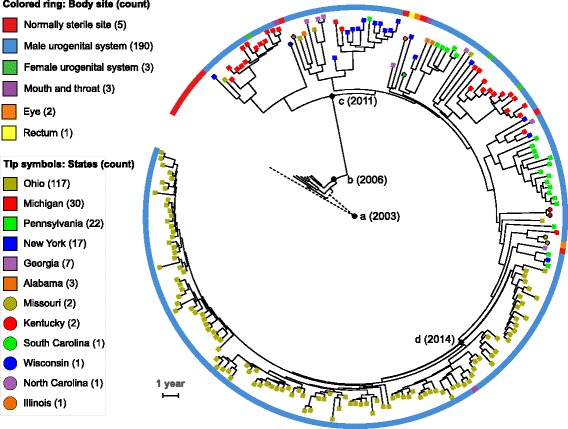
Time-measured Bayesian phylogeny U.S. NmNG urethritis clade. The analysis included 204 isolates belonging to the clade, with nine closely related invasive isolates used as an outgroup. Isolates are marked according to the state in which they were isolated (symbols on branch tips), and body site from which they were isolated (colored ring). Counts in the key refer to isolates in the U.S. NmNG urethritis clade. Nodes mentioned in the text are marked with black circles and labeled with the estimated year: (**a**) the root; (**b**) divergence between urethritis clade and NmC isolates, (**c**) the U.S. NmNG urethritis clade; and (**d**) the clade comprising solely isolates from Columbus, OH. The two branches originating at the root (node “a”) are dashed to indicate that inferred mutational events on the branch leading to the outgroup cannot be oriented with respect to time. The scale bar represents 1 year

The 209 isolates belonging to the U.S. NmNG urethritis clade were collected in 13 different states between 2013 and 2016 (Additional file 1). To estimate the evolutionary timeline of this clade, the relationships among the clade and closely related isolates was inferred using BEAST v1.8.3 [27], which models evolution with a molecular clock (Fig. 2). This analysis included 204 of the 209 U.S. NmNG urethritis clade isolates, with five excluded because high-quality read data was not available (see methods). To provide an outgroup, the analysis included the eight closest relatives to the U.S. NmNG urethritis clade from the CDC strain collection. An additional closely related NmC isolate with a complete genome sequence (M21273) was included as a reference for sequence alignment.

The U.S. NmNG urethritis clade was estimated to have diverged from the eight closely related invasive disease isolates at 2006.3 (Fig. 2, node b; 95% HPD interval = [2005.8, 2006.7]). The U.S. NmNG urethritis clade was confidently identified as a separate branch from the closely related invasive disease isolates (Bayesian posterior clade probability = 1), with its most recent common ancestor estimated to have existed at 2011.4 (Fig. 2, node c; 95% HPD interval = [2010.8, 2012.0]). Within the urethritis clade, the vast majority of isolates from Columbus, Ohio (112/117) belonged to a strongly supported clade with no isolates from other states (Fig. 2, node d; posterior probability = 1), and its most recent common ancestor was estimated to have existed at 2014.1 (95% HPD interval = [2013.7, 2014.3]). In addition to 111 isolates obtained from urethral swabs at the Columbus clinic, this clade included one isolate obtained from an oral swab at the same clinic.

Isolates submitted from other states were phylogenetically dispersed (Fig. 2). Three of the five isolates from normally sterile sites of patients with invasive meningococcal disease were isolated in states that also submitted isolates from the urogenital system, yet the isolates from normally sterile sites were not closely related to the urogenital isolates from the same state.

### The U.S. NmNG urethritis clade has few changes in gene content

Using the same set of isolates as shown in Fig. 2, gene content variation among 204 U.S. NmNG urethritis clade isolates and the eight most closely related invasive meningococcal disease isolates were examined to identify gain or loss of genes. Consistent with previous findings, all 204 U.S. NmNG urethritis clade isolates lacked the capsule synthesis genes *cssA*, *cssB*, and *cssC,* and retained only a partial (“incomplete”) *csc* gene sequence. Only one other coding sequence, NEIS1859 (*autA*, autotransporter A), was absent in all members of the urethritis clade but present in more than four of the closely related invasive meningococcal disease isolates. NEIS1859 was reported as “incomplete” in the U.S. NmNG urethritis clade genome assemblies, but it was reported as allele 326 in the seven closely related NmC genomes and missing in the closely related genome of NmNG M16917. This locus was confirmed to contain IS*1301* in all members of the U.S. NmNG urethritis clade based on the mapping of paired reads to both IS*1301* and NEIS1859 using ISMapper v1.2 [28].

No gene was contained by more than two of the 204 U.S. NmNG urethritis clade genomes without also being contained by at least one of the eight closely related outgroup genomes. The *nadA* (NEIS1969) locus was disrupted by IS*1301* in the eight closely related invasive meningococcal disease isolates and the reference genome M21273 (allele 29), but the IS element was absent from the *nadA* locus in 192 of the 204 genomes belonging to the urethritis clade (Additional file 1).

### Gene flow from *N. gonorrhoeae* has affected the U.S. NmNG urethritis clade

The acquisition of genetic material through horizontal gene transfer was explored using ClonalFrameML [29] to identify regions of the core genome alignment where divergent DNA sequences were incorporated into the genomes of these isolates. In total, 581 recombination events were identified along the BEAST-generated tree (Fig. 2; Additional file 3), with estimated parameters of R/theta = 0.41 (ratio of recombination events to point mutation events), nu = 3.7% (mean polymorphisms per recombinant tract), and delta = 1253 bp (mean recombination tract length). Of these 581 recombination events, 64 were on the branch leading to the outgroup (Fig. 2, dashed branches from root node a), 18 on branches leading to the closely related invasive meningococcal disease isolates (Fig. 2, solid branches basal to node b; total length 3.96 × 10^− 5^ substitutions per site), eight on the branch leading to the root of the U.S. NmNG urethritis clade (Fig. 2, branch between nodes b and c; length 1.51 × 10^− 5^), and 491 within the U.S. NmNG urethritis clade (Fig. 2, node c; total length of 8.95 × 10^− 4^).

Recombinant sequences were used as BLAST queries to search the NCBI RefSeq Genomic DNA library, identifying matches for 515 of the sequences (Additional file 3). The remaining 66 small recombination events included no more than 39 nucleotides each, representing 271 substitutions in total. Within the U.S. NmNG urethritis clade, 429 recombinant sequences matched RefSeq genomes, of which 145 were inferred to have originated from a species other than *N. meningitidis*. *N. gonorrhoeae* was the inferred source for 138 recombination events, while *N. lactamica* and *N. cinerea* were each the inferred source for two events. *N. polysaccharea* was the inferred source for one recombinant sequence, and two recombinant sequences matched the genomes of unspecified *Neisseria spp*. Of the remaining 284 recombinant sequences with hits, 272 were most similar to *N. meningitidis* sequences, while 12 had ambiguous origins. Along the branch leading to the most recent common ancestor of the U.S. NmNG urethritis clade, three of the eight recombinant sequences with matches to RefSeq genomes were inferred to have originated in *N. gonorrhoeae,* with three originating from other *N. meningitidis* genomes and two being of ambiguous origin. Along the branches leading to the eight invasive meningococcal disease isolates not belonging to the U.S. NmNG urethritis clade, only one of 17 recombinant sequences with matches in the RefSeq database was inferred to have originated from another species, *N. gonorrhoeae*, affecting only a single genome (M16917).

The ancestors of the U.S. NmNG urethritis clade were inferred to have acquired *N. gonorrhoeae* alleles in three genomic regions containing a total of 7638 bp and eight genes (Additional file 4). One recombination event covered 42% of the acetylglutamate kinase gene *argB* (NEIS1038) and changed 3.4% of nucleotides, resulting in 100% sequence identity to *N. gonorrhoeae*. A second recombination event covered 100% of the nitrite-reductase gene *aniA* (NEIS1549) and 96% of the nitric oxide reductase gene *norB* (NEIS1548)*,* and changed 4.8% of nucleotides, resulting in 99.8% identity to *N. gonorrhoeae*. A third recombination event covered 85% of the 2-C-methyl-D-erythritol 4-phosphate cytidylyltransferase gene *ispD* (NEIS1442) and all of the adjacent four loci (NEIS1443–1446): the DNA polymerase III subunit epsilon gene *dnaQ*, a homolog of the lysophospholipid transporter gene *lplT*, a homolog of a cbb3-type cytochrome oxidase maturation protein *fixS,* and a conserved hypothetical protein. This recombination event changed 3.7% of nucleotides, resulting in a sequence with 98.5% identity to *N. gonorrhoeae.*

**Fig. 3:**
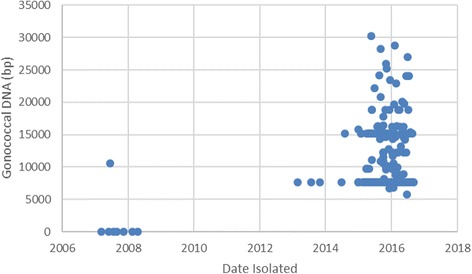
Gonococcal DNA content of U.S. NmNG urethritis clade isolates and closely related invasive isolates. Sequence change is measured relative to M21273 and plotted with respect to the date of isolation. Isolates collected before 2012 are not part of the U.S. NmNG urethritis clade

Recombination events identified within the U.S. NmNG urethritis clade (including the most recent common ancestor) totaled 864,056 base pairs (bp), but because some events overlapped, only 556,610 bp of the alignment were affected by at least one recombination event. Cross-species recombination events affected 274,420 bp, with 262,267 bp being affected by events inferred to involve *N. gonorrhoeae* DNA*.* The total length of DNA sequences inferred to have originated from *N. gonorrhoeae* varied substantially among the 204 members of the U.S. NmNG urethritis clade (Fig. 3, Additional file 1). While several of the isolates contained only the gonococcal DNA present in the common ancestor (7638 bp in three regions), others had as much as 30,208 bp. Furthermore, others had as little as 5713 bp due to acquisition of non-gonococcal DNA sequences during subsequent recombination events at the loci that had contained gonococcal sequences in the most recent common ancestor. Gonococcal DNA was identified in 268 of the 2256 loci annotated by PubMLST for M21273, covering at least 50% of the locus in at least one genome. Similarly, gonococcal DNA was identified in 249 of the 2045 loci annotated by Prokka (Additional file 4).

Several loci were impacted by multiple recombination events (Additional file 4). For the three stretches of gonococcal DNA contained by the most recent common ancestor of the U.S. NmNG urethritis clade (totaling 7638 bp and including eight PubMLST loci), two regions were involved in subsequent recombination events that affected five PubMLST loci. The *aniA* and *norB* genes (NEIS1458 and NEIS1459) were involved in three subsequent recombination events. One event (affecting seven genomes) introduced meningococcal-like sequences for 100% of *aniA* and 64% of *norB*. The other two events affected a single genome each and covered short portions (< 50%) of both *aniA* and *norB*; one sequence was inferred to have originated from *N. meningitidis* and the other was inferred to have originated from *N. gonorrhoeae*. Despite this diversity of DNA sequences at *aniA* and *norB,* all isolates within the U.S. NmNG urethritis clade contained full length open reading frames of both genes. This occurred even though the eight closely related invasive disease isolates and the two other NmNG CC11 urethral isolates all included a frameshift mutation leading to a premature stop codon in *aniA* (allele 1) and the eight closely related invasive meningococcal disease isolates also had a premature stop codon in *norB* (allele 149) [8] .

Likewise, at PubMLST loci NEIS1442–1444 (homologous to *ispD, dnaQ*, and *lplT*, respectively), the gonococcal DNA acquired by the U.S. NmNG urethritis clade was replaced by sequences most similar to *N. meningitidis* sequences during three recombination events that affected six genomes. One additional recombination event occurred at NEIS1444, with a *N. gonorrhoeae* genome providing the best match. These subsequent recombination events affected no more than 450/1311 bp of the *lplT* homolog, and did not affect NEIS1445 (FixS/CcoS gene family) and NEIS1446, which had also been converted to the gonococcal alleles during the initial recombination event.

### A gonococcal antimicrobial resistance determinant was detected in one isolate

The isolates collected for this study were examined for the presence of sequence variants that are associated with reduced antimicrobial susceptibility in *N. gonorrhoeae.* No variants were detected at amino acid or nucleotide positions associated with tetracycline, spectinomycin, quinolone, cephalosporin, or macrolide resistance. Two non-CC11 isolates (M38608 and M38636, from rectal and vaginal swabs, respectively) contained meningococcal *mtrR* sequences that are associated with elevated azithromycin MICs of 2 μg/mL according to NG-STAR [30]. In contrast, one isolate from the U.S. NmNG urethritis clade collected in 2015 (M38896) had a gonococcal-like *mtrR* sequence (allele type 39) that is also associated with elevated azithromycin MICs of 2 μg/mL. This allele conversion occurred as part of a 7362 bp recombination event (Additional file 3), which contributed to this isolate having the greatest total length of gonococcal-like DNA sequences among all isolates in the U.S. NmNG urethritis clade, with 30,208 bp.

### Genes for *N. meningitidis* serogroup B vaccine antigens are common among the U.S. NmNG urethritis clade

The 209 isolates belonging to the U.S. NmNG urethritis clade all encode peptides that are targeted by protein-based vaccines developed to protect against serogroup B meningococcal disease. All 209 isolates encode NhbA peptide 20. Two isolates have a stop codon in the *fHbp* coding sequence (alleles 1249 and 1250), while 207 encode FHbp peptides belonging to subfamily B/variant 1 (peptides 896, 456, and 915 encoded by alleles 1127, 1237, and 1146, respectively). NadA-2/3.2 is encoded by 193 genomes, while it is disrupted by IS*1301* in 13 genomes, and by a premature stop codon in three genomes. The PorA type is P1.5–1,10–8 in 198 genomes, of which four had an internal stop codon prior to variable region 2. PorA type P1.5–1,10–1 was present in nine genomes and P1.5–1,10–22 and P1.17,9 were each present in one genome (Additional file 1).

A small clade of NmNG urethral isolates was also identified in sublineage 11.2 (Fig. 1, blue star). These two isolates encoded NhbA peptide 20, but were predicted not to express FHbp or NadA due to a stop codon in the *fHbp* allele 669 and a IS*1301* element in the *nadA* gene. Both encode PorA type P1.5–1,10–1.

## Discussion

### The U.S. NmNG urethritis clade is an emerging pathogen

Occasional reports of *N. meningitidis* urethritis, and even small clusters of *N. meningitidis* urethritis, in the United States date back to the 1940s [2, 4]. Reported cases have been attributed to diverse lineages [31], many of which are commonly associated with invasive meningococcal disease. However, the U.S. NmNG urethritis clade within lineage 11.2 is a new paradigm; the clade is primarily cultured from cases of male urethritis and only 5 of the 209 known isolates in the clade originated from cases of invasive disease (Figs. 1 and 2).

Increased numbers of *N. meningitidis* urethritis isolates have been received by the CDC since 2015, as awareness was raised by the identification of clusters of *N. meningitidis* urethritis at GISP sentinel clinics in Columbus, Ohio and Oakland County, Michigan [11]; these reports were followed by a request for isolates through the Epidemic Information Exchange (Epi-X), a secure communications network for public health officials. Among the collection of 209 urogenital and rectal isolates received by the CDC in 2015–2016, we identified two groups of closely related isolates from urethritis cases: the above-mentioned U.S. NmNG urethritis clade, and a pair of NmNG isolates constituting a second lineage 11.2 clade (M39848, M40415; Fig. 1, blue star). Both of these clades of urethral isolates from the United States were more closely related to invasive disease isolates from the U.S. than to the non-U.S. urogenital isolates listed by Tzeng et al. [8], indicating their separate evolutionary origins from among lineage 11.2 populations associated with invasive meningococcal disease. This repeated observation of phylogenetically separated urogenital isolates may indicate that lineage 11.2 has a greater tendency to colonize the urethra and cause urethritis than do other *N. meningitidis*. The non-lineage 11 isolates from the male urogenital system studied were not closely related to each other or to isolates from the rectum or the female urogenital system, suggesting that these lineages do not commonly cause disease at those body sites. While the prevalence of non-lineage 11 strains among urogenital and rectal infections cannot be estimated from the isolate collection described here, many of these isolates were submitted from the same states as the U.S. NmNG urethritis clade. This suggests that they are a less common cause of urogenital infections than the U.S. NmNG urethritis clade, even in the geographic locations where they were collected.

Overall, these results indicate that the U.S. NmNG urethritis clade is unusual among *N. meningitidis* for being recovered from a large number of urethritis cases. Having likely diverged from other *N. meningitidis* after 2006 and spread to at least 13 states since the most recent common ancestor existed in 2011 (Fig. 2), this clade of *N. meningitidis* appears to be an important emerging urogenital pathogen.

### The U.S. NmNG urethritis clade can cause invasive meningococcal disease

Capsule expression contributes to virulence during invasive meningococcal disease [8, 19], so the disruption of the capsule locus in the U.S. NmNG urethritis clade was expected to limit the risk of invasive meningococcal disease from this clade. However, by screening genome sequences obtained through surveillance systems for invasive meningococcal disease, we identified five nongroupable isolates from invasive disease cases that belonged to the U.S NmNG urethritis clade, including the two earliest representatives, from 2013. Invasive disease from NmNG strains is generally rare [32] but some medical conditions and treatments are associated with higher rates of NmNG infection (e.g. Eculizumab [33]). The contribution of medical conditions to the risk of invasive disease involving the U.S. NmNG urethritis clade cannot be assessed with the information available for these five cases.

Interestingly, the four states in which these five invasive meningococcal disease isolates were collected were not the same as the states where the greatest number of *N. meningitidis* urethritis isolates were collected (Additional file 1, Fig. 2). Furthermore, the two invasive disease isolates from Georgia and one from New York were not most closely related to the urogenital isolates from the respective states. The identification of invasive meningococcal disease isolates from the U.S. NmNG urethritis clade does show that this lineage has the potential to cause invasive infections and its prevalence should be monitored as a potential contributor to invasive meningococcal disease. However, the frequent detection of the clade in a geographic locality does not currently appear to indicate an elevated risk of invasive disease from this pathogen. Increased understanding of the relationship between *N. meningitidis* urethritis and invasive disease can be facilitated by conducting active surveillance for both invasive disease and urogenital infections in overlapping areas.

Urethral colonization has been proposed to contribute to invasive meningococcal disease among MSM [9, 10], and the ability to reduce nitrite has been observed among isolates collected from invasive cases among MSM as well as from *N. meningitidis* urethritis/proctitis isolates, pointing to a physiological requirement for colonization of and transmission from the urethra [7, 8]. There is currently no evidence that the U.S. NmNG urethritis clade is substantially contributing to urethritis [11] or invasive disease [18] among MSM in the United States, although two of the five invasive meningococcal disease case-patients infected by the U.S. NmNG urethritis clade were MSM. Notable outbreaks of invasive meningococcal disease among MSM involved serogroup C (NmC) CC11 strains [7, 15–18], while the CC11 urethritis isolates evaluated in this study were all nongroupable and mostly isolated from heterosexual populations [12]. Furthermore, the urogenital isolates examined in this study were not most closely related to the NmC isolates collected from the outbreaks of invasive meningococcal disease among MSM in the United States since 2010 (Fig. 1). Attempts to identify lineages that are associated with specific diseases can be confounded by bacterial population structure, particularly geographic and temporal variation, so identifying the role, if any, of urethral colonization in the spread of invasive meningococcal disease among MSM will require a targeted evaluation of transmission patterns among this demographic group.

### Vaccines may protect against U.S. NmNG urethritis clade infections

The identification of invasive disease cases involving the U.S. NmNG urethritis clade indicates the value of further research into whether the new protein-based vaccines developed to protect against serogroup B meningococcal disease could provide protection against infection from this nongroupable clade and other CC11 organisms [34]. The first requirement for vaccine protection is that the pathogen encodes the genes that produce the antigens targeted by the vaccine. Both the MenB-4C and MenB-FHbp vaccines contain FHbp components with the same subfamily/variant classification as the FHbp peptide encoded in most of the U.S NmNG urethritis clade genomes [35, 36]. For the remaining components of the MenB-4C vaccine, all clade genomes encode NhbA and most encode NadA, which may contribute to immunogenicity. However, the PorA peptide does not match the vaccine component (type 4 in variable region 2), suggesting that it will not contribute to immunogenicity against this clade [37]. For these vaccines to provide protection against the U.S. NmNG urethritis clade, the peptides also are required to be expressed. Preliminary data (Tzeng Y-L, Stephens DS et al. unpublished) indicates FHbp is highly expressed on the surface of clade isolates.

Vaccines developed to protect against serogroup B invasive disease could also protect against urethritis. A recent case-control study showed that recipients of a meningococcal outer-membrane vesicle (OMV) vaccine had a lower likelihood of *N. gonorrhoeae* infection than had their unvaccinated peers [38]. The MenB-4C vaccine contains the same OMVs.

### Genome-based surveillance can rapidly identify emerging pathogens

Surveillance programs were essential for identifying and collecting the isolates constituting the U.S. NmNG urethritis clade, both from urethritis (GISP) and from invasive meningococcal disease cases (Active Bacterial Core surveillance and Enhanced Meningococcal Surveillance). While surveillance programs process a large number of specimens, their methods are defined for the identification of specific pathogens from specific clinical specimens. Therefore, they are not necessarily effective at identifying new emerging pathogens in these specimens. The increased detection of *N. meningitidis* among urethritis specimens was originally noticed at two GISP sentinel clinics due to discrepancies in two tests used for the diagnosis of *N. gonorrhoeae* infections -- specimens containing gram-negative intracellular diplococci had negative results on a NAAT targeting gonococcal DNA [11]. This process illustrates the value of conducting confirmatory tests for pathogens during surveillance activities. Whole genome sequencing not only allowed confirmation of *N. meningitidis* but also enabled high-resolution typing of this organism.

### Whole genome sequencing reveals the evolutionary process of pathogen emergence

Whole genome sequencing not only allows for the confirmation of a suspected pathogen, high-resolution typing, and rapid identification of unexpected pathogens, but also holds promise for detecting novel pathogens by sequencing clinical specimens, through either amplicon deep sequencing or metagenomic approaches. Such microbiome analyses can be particularly powerful when disease etiology is unclear.

This study demonstrates the power of whole genome sequencing to provide precise information about the evolutionary origin of an emerging pathogen and identify genetic changes that could contribute to its novel pathogenicity. The U.S. NmNG urethritis clade is distinct from the lineage 11.2 strains responsible for outbreaks of invasive meningococcal disease among MSM in the United States and Europe as well as from the published isolates recovered from urethritis cases in Europe. Not only is the clade nongroupable while the lineage 11.2 isolates associated with the MSM outbreaks and many prior urethritis cases have been serogroup C, but the most closely related NmC isolates were not recovered from the recent outbreaks among MSM (Fig. 1). Instead, several invasive disease isolates not from MSM from 2007 and 2008 were identified as being closely related to the urethritis isolates.

The identification of closely related invasive disease isolates allowed the U.S. NmNG urethritis clade origin to be placed between late 2005 and early 2012. Several potentially critical genetic changes occurred during this period, but the timing of individual changes remains unresolved due to the lack of isolates that diverged from the clade during this period.

While the gain of novel genes appears not to have contributed to the origin of the U.S. NmNG urethritis clade, the loss of multiple genes could have had a critical impact on its pathogenic properties, which has been observed with other bacterial pathogens [39]. The disruption of the capsule locus in the U.S. NmNG urethritis clade results in a nongroupable phenotype, which is similar to *N. gonorrhoeae* and may contribute (e.g. through enhanced mucosal attachment) to the high frequency with which this clade causes urethritis [8, 12]. This deletion completely removed the sialic acid capsule biosynthesis genes (*cssA-C*) and part of the serogroup C capsule polymerase gene (*csc*). This configuration appears to be stable among all of the isolates in this clade, as the respective genes are absent from the genome assemblies, but the exact structure of the capsule locus cannot be determined from short-read data due to the presence of a IS*1301* insertion [8]. Elimination of capsule expression may play a role in facilitating urethral colonization, as indicated by the nongroupable phenotype in both the U.S. NmNG urethritis clade and the other two ST-11 urethral isolates from this study (Fig. 1, blue star), in which capsule expression was disrupted by a premature stop codon caused by a frameshift mutation in the capsule translocation gene *ctrF.*

The only other gene that was disrupted during the early evolution of the urethritis clade was *autA*. The AutA protein is immunogenic and may influence meningococcal autoaggregation and biofilm structure [40, 41]; however, its expression is not a characteristic difference between *N. meningitidis* and *N. gonorrhoeae*, since *autA* is phase-variable in both species [41, 42]. A 3.7 kb recombination event introduced gonococcal DNA at the locus responsible for nitrite-dependent anaerobic growth (NEIS1548-NEIS1549), replacing *aniA* and most of *norB*, while eliminating internal stop codons in both genes [8]. The reconstitution of the *aniA* ORF can enable nitrite-dependent anaerobic growth in *N. meningitidis*, and several isolates from the U.S. NmNG urethritis clade have been shown to possess this capability [8]. Nitrite-dependent anaerobic growth has been proposed to be an important contributor to urethral colonization by some *N. meningitidis* [7], and it is a fundamental distinction between *N. gonorrhoeae* and *N. meningitidis* in general [43].

Another acquisition of gonococcal DNA covered a 3.1 kb region that included five genes (NEIS1442-NEIS1446). This included a potential coding region for a 61-residue protein of the FixS/CcoS family (NEIS1445), which is essential for the maturation of cbb3-type cytochrome oxidase [44]. Cytochrome cbb3 oxidase reduces oxygen as an alternative pathway to AniA (NirK)-mediated reduction of nitrite in *Neisseria*, but it can also act as an electron donor to AniA in *N. gonorrhoeae* [45]. The other ORFs impacted by this recombination event show homology to an isoprenoid synthesis gene (*ispD*), the DNA polymerase subunit epsilon (*dnaQ*), and a lysophospholipid transporter (*lplT*). Subsequent acquisitions of meningococcal DNA at this locus did not include the FixS/CcoS family coding sequence, even as they did include the *ispD* and *dnaQ* homologs as well as one-third of the *lplT* homolog. This reversion to meningococcal sequences at *ispD*, *dnaQ,* and *lplT* among urethral isolates may indicate that the gonococcal version of these genes do not contribute to urethral colonization or urethritis pathogenicity, but that the FixS/CcoS family coding sequence or its upstream sequences do. If so, the contribution may not be due to changes in the FixS/CcoS family protein, which only acquired a single amino acid polymorphism; instead, the contribution of gonococcal FixS may result from gene expression changes, which could be mediated by upstream sequences or the eleven synonymous polymorphisms in the gene [46].

Acquisitions of gonococcal DNA have continued following the establishment of the U.S. NmNG urethritis clade (Fig. 3, Additional file 4). These additional gonococcal sequence variants are only present in a fraction (< 30) of the isolates collected during surveillance. Continued surveillance and isolate collection will enable tracking of whether these gonococcal sequences become more frequent in urethritis-associated *N. meningitidis.* Of note, a single isolate from the U.S. NmNG urethritis clade contained an *mtrR* promoter and coding sequence that had been associated with decreased susceptibility to azithromycin in *N. gonorrhoeae*. This isolate was collected in 2015, and this *mtrR* sequence was not detected in any isolates collected in 2016.

### Niche invasion and speciation

*N. gonorrhoeae* has been proposed to have originated as a clone of the *N. meningitidis* progenitor that switched from primarily colonizing the nasopharynx to primarily colonizing the urogenital system [20]. While each species is capable of colonizing the primary habitat of the other, ecological separation could contribute to the rarity of gene flow between these two species [20, 21], with either physical separation or incompatible adaptations being responsible. Molecular mechanisms could also contribute to the genetic isolation of the species, including the binding-specificity of the ComP protein with species-specific DNA uptake sequences, the activity of species-specific restriction endonucleases, and the tendency of sequence divergence to inhibit homologous recombination [47]. The detection of multiple gonococcal DNA sequences in recombinant genomic regions within the U.S. NmNG urethritis clade suggests that one or more of these barriers may have been weakened, and gene flow from *N. gonorrhoeae* to the U.S. NmNG urethritis clade has contributed to the emergence of this clade as a urogenital pathogen.

Orogenital contact appears to play a role in the initiation of urethral colonization, as most patients in the initial Columbus study reported oral sex in the month before diagnosis [12]. Furthermore, one isolate in the U.S. NmNG urethritis clade (Fig. 2) was obtained from an oral swab provided by a sexual partner of a meningococcal urethritis patient. Additional isolates belonging to the U.S. NmNG urethritis clade were recovered from the rectum and female urogenital system, suggesting that it may also be able to colonize these sites. However, genital-to-oral transmission or genital-to-genital transmission have not been investigated, leaving open the possibility that urethral colonization is an evolutionary dead-end for *N. meningitidis*. The importance of urethral colonization to the natural history of the U.S. NmNG urethritis clade, the risk of this clade causing urogenital disease in females, and the transmission dynamics of the clade in both a urogenital and nasopharyngeal niche are areas of future study.

## Conclusions

A novel clade of *N. meningitidis* has emerged in the past decade as an important male urethritis-associated pathogen. During this time, the clade has spread among several states and gained gonococcal genetic material, potentially facilitating urethral colonization. However, the U.S. NmNG urethritis clade is also capable of causing invasive meningococcal disease, and the risk posed as a urethral pathogen and invasive meningococcal disease pathogen is still unclear. To date the clade has not acquired common gonococcal antimicrobial resistance factors. However, to the extent that the ecology and clinical presentation of the NmNG urethritis clade mirrors that of gonococcal infections, the evolutionary forces that resulted in high rates of antimicrobial resistance among *N. gonorrhoeae* may lead to the same result among these *N. meningitidis*. Assessment of disease risk and the development of control strategies will be facilitated by a greater understanding of the prevalence of colonization by the U.S. NmNG urethritis clade and the modes of transmission. The results reported here illustrate the value of applying whole genome sequencing to disease surveillance in order to quickly identify and characterize emerging pathogens and track their evolution.

## Methods

### Isolate collection and characterization

Isolates within the CDC *N. meningitidis* strain collection that belonged to the U.S. NmNG urethritis clade were identified using a SNP-based parsimony phylogeny (described below) to compare 343 CC11 *N. meningitidis* isolates with available genome sequences to the isolates described by Bazan et al. [12]. The 343 CC11 isolates were collected in the USA between 1994 and 2016; 304 were associated with invasive cases and 39 were not. Isolates were considered closely related to the U.S. NmNG urethritis clade if they were at least as closely related to the clade as M16917, which had previously been identified as the most closely related isolate [12]. These isolates are described in Additional file 1.

Following the initial report of clusters of meningococcal urethritis [11] the CDC requested that state and local public health departments report any urethritis cases that tested both GNID positive and NAAT negative for *N. gonorrhoeae* and submit associated isolates to the CDC for characterization. The request was made on February 17, 2016 through the Epidemic Information Exchange (Epi-X) [48], CDC’s system for rapid and secure exchange of public health information between CDC and state and local health departments. GISP isolates were collected as described previously [12]. Urogenital and rectal isolates collected after January 1, 2015 and received before September 30, 2016 were included in this analysis, for 209 isolates (Table 1, Additional file 1). Three additional NmNG invasive disease isolates from 2014 and 2015 were selected from the CDC strain collection, sequenced, and included in the analysis. Isolates were confirmed to be *N. meningitidis* using species-specific real-time PCR, while serogroup was identified using slide agglutination and confirmed using real-time PCR [49].

This study also included meningococcal genome sequences previously published on PubMLST.org [50] and NCBI BioProjects PRJNA400487 and PRJNA378732, as depicted in Additional file 1. These included genomes of 41 non-U.S. CC11 isolates (21 from the male urogenital system, 12 from female urogenital system, and eight from MSM invasive cases) described by Tzeng et al. [8], 4 NmNG CC11 urethritis isolates from Indiana [8, 13] and 74 genomes from U.S. CC11 invasive disease isolates (31 from four invasive meningococcal disease outbreaks among MSM from 2009 to 2016 [16–18, 26] and 43 from invasive cases in the same geographical region as the outbreaks among MSM). FAM18 [51] and an isolate from the serogroup W Hajj-related outbreak in 2000, M07149 [52], were included as phylogenetic reference genomes.

If the body site from which an isolate was collected was undocumented, then the body site was inferred from the disease associated with the isolate. Isolates were grouped by body site as follows: male urogenital system (urine, urethral swab, and urethritis-associated body site), female urogenital system (vaginal swab, cervical swab, and vaginal discharge-associated body site), mouth and throat (oral swab and oropharyngeal swab), normally sterile site (blood, CSF, joint fluid, or specimens associated with meningitis or septicemia), eye (eye swab), and rectal (rectal swab).

### Genome sequencing and characterization

Whole genome sequence data were generated as 250 bp paired-end reads using either an Illumina HiSeq 2500 or MiSeq and submitted to NCBI under BioProject PRJNA324131. DNA was extracted from plated isolates using ArchivePureTM DNA purification kit (5 prime, Gaithersburg MD, USA), sheared to 600 bp using a Covaris LE220 focused ultrasonicator (Covaris Inc., Woburn, MA), and libraries were constructed using the NEBNext Ultra DNA library preparation kit (New England Biolabs, Ipswich, MA, USA). Reads were trimmed with CutAdapt v1.8 [53] and assembled with SPAdes v3.7 [54]. Locus annotations were obtained from both PubMLST [50] and Prokka v1.11 [55]. Gene content variation was examined using the PubMLST.org GenomeComparator tool. Multilocus Sequence Typing (MLST) alleles and outer membrane proteins were identified by searching assembled genomes with PubMLST allele lists using BLAST v2.2.30 [56]. Porin A (PorA), Porin B (PorB), and Ferric enterobactin transport (FetA) types are defined by their respective variable regions [57]. *Neisseria* adhesin A (NadA) is identified by variant and peptide ID, as suggested by Bambini et al. [58]. Disruption of NadA by IS*1301* was inferred if portions of the NadA gene were identified in two genomic locations, each adjacent to a sequence from IS*1301*. Genome assemblies downloaded from PubMLST were not assessed for IS*1301* insertions. Both *Neisserial* Heparin Binding Antigen (NhbA) and Factor H binding protein (FHbp) are identified by their PubMLST peptide identifiers.

### Identification of variants associated with *N. gonorrhoeae* antimicrobial resistance

To determine if any of the meningococcal genomes contained sequences related to gonococcal-specific resistance, raw reads were mapped to the *Neisseria gonorrhoeae* FA19 reference genome using CLC Genomics Workbench (CLC bio, Aaarhus, Denmark), and variants were called using the fixed ploidy variant detection model. Variants associated with resistance or reduced susceptibility to penicillin (*penA* D354-insertion; *ponA* L421P), tetracycline (*rpsJ* V57 M/L/Q), spectinomycin (*rpsE* V25-deletion, K26E), quinolones (*parC* S87R/N/I; *gyrA* D95G/A/N, S91F), macrolides (*mtrR* promoter region A-deletion, interspecies mosaic alleles; *mtrR* G45D), and cephalosporins (*penA* mosaic XXXIV; *ponA* L421P; *porB* A121N) were examined. Macrolide resistance in *N. gonorrhoeae* is also associated with variants in 23S rRNA (C2611T and A2059G), and variants were examined using a signal ratio analysis as previously described [59]. Sequences from isolates exhibiting known gonococcal variants were subsequently subjected to BLAST [56] and *Neisseria gonorrhoeae* Sequence Typing for Antimicrobial Resistance (NG-STAR) [30] analyses for further characterization.

### Phylogenetic analysis

SNP-based parsimony phylogenies (Fig. 1) were inferred from genome assemblies by the kSNP v3.0 pipeline [60]. kSNP examines all 25 bp sequences in each genome (k-mers) and reports core single nucleotide polymorphisms (SNPs) for all sets of k-mers that differ only at the central (i.e. 13th) base, and are present in all genomes. The collection of identified SNPs was then analyzed by RAxML v7.3 [61] to identify the most parsimonious phylogeny.

A time-measured phylogeny (Fig. 2) was inferred using BEAST v1.8.3 [27] based on a recombination-masked core genome alignment of the 213 U.S. NmNG urethritis clade and closely related isolates. The alignment was generated by first calling consensus nucleotide sequences for each genome from Illumina short read data using the Snippy v3.1 pipeline [62], which both mapped reads and called consensus nucleotides based on the complete, circular genome sequence of the closely related isolate M21273 as a reference. Genomes obtained from PubMLST as assemblies cannot be used in this analysis, since this requires read data. One isolate was removed from the alignment due to low coverage of the reference genome (M39066, 83% coverage), and the remaining isolates each exceeded 93% coverage. Columns with gaps or ambiguous bases were masked, retaining a 1,972,278 bp core alignment (89% of M21273) with 27,271 polymorphic sites. Recombinant tracts were masked using Gubbins v1.4.1 [63], which identifies recombination based on the chromosomal clustering of mutations that occur on the same branch of a phylogenetic tree. Gubbins was run with up to 10 iterations and a minimum of 5 SNPs in a recombination tract, identifying 308 recombination events on 115 branches with 0.155 recombination events per point mutation events and 16.48 polymorphisms introduced by recombination per polymorphism from mutation, leaving 1343 polymorphic sites. To evaluate the impact of recombination masking on the ability to predict the date that an isolate was collected, the correlation between isolation date and root-to-tip distance was established with TempEst v1.5 [64] based on maximum likelihood phylogenies inferred from the masked and unmasked alignments (r = 0.76 and 0.52, respectively) using RAxML v8.2.4 with the “GTRGAMMAX” substitution model and “autoMRE” bootstrap stopping algorithm [61]. The recombination-masked Gubbins alignment was analyzed by BEAST v1.8.3 [27] to perform Bayesian inference of parameters in the HKY substitution model, using a chain length of 10^8^; the estimated clock rate was 3.03 × 10^− 6^ substitutions per year (Effective Sample Size = 751) and estimated kappa was 7 (Effective Sample Size = 8689).

Recombinant regions were identified on the BEAST-generated tree using ClonalFrameML 1.25 [29], which uses a maximum likelihood phylogenetic model to identify recombination events that introduced clusters of polymorphisms on the chromosome. This method is known to undercount recombination events that introduce a small number of polymorphisms [29]; however, ClonalFrameML infers the genome sequence of all ancestors in the phylogenetic tree and can identify repeated recombination events that occurred at the same locus. The percentage of nucleotides changed by a recombination event was calculated as the percentage of nucleotides in the recombinant region that differed between the recombinant node and its parent node in the tree, excluding nucleotide positions that had been masked because of gaps or ambiguous bases. The recombinant sequences identified by ClonalFrameML were BLAST-searched against the NCBI RefSeq Genomes database (retrieved February 1, 2017) to identify potential sources of recombination [56]. Recombinant sequences were inferred to have originated from a different species if they matched a sequence from that species with at least 0.5% greater sequence identity than any match to *N. meningitidis* (including the outgroup sequence M21273 and the inferred ancestral sequence from ClonalFrameML), with the remaining recombinant sequences being identified as having ambiguous origin.

Phylogenetic trees were drawn by iTOL [65], and analyses made use of SciPy v0.18 [66] and BioPython v1.68 [67].

The findings and conclusions in this report are those of the authors and do not necessarily represent the official position of the Centers for Disease Control and Prevention.

## Additional files

Additional file 1: List of 353 isolates included in analyses of the U.S. NmNG urethritis clade, including sequence accession information, and provenance, serogroup, molecular typing, and impact of recombination events where available. (XLSX 53 kb)

Additional file 2: Depiction of isolate collection included in each analysis. Regions are not to scale. All Isolates are included in phylogenetic analysis for Fig. 1 (*n* = 353). The bases for inclusion are listed in the margins. Isolates belonging to the U.S. NmNG urethritis clade are outlined, and isolates included in Fig. 2 are identified with diagonal hatching. Cross (+) identifies isolates that were excluded from analysis in Fig. 2 due to inappropriate data format. Asterisk (*) marks an isolate that was excluded from analysis in Fig. 2 due to insufficient data quality. (TIFF 127 kb)

Additional file 3: Homologous allele replacements identified by ClonalFrameML, including gene annotations and the results of searching the BLAST RefSeq genomic database. The “Dictionary” worksheet defines the columns used in the “Recombination events” worksheet. (XLSX 230 kb)

Additional file 4: Loci affected by homologous allele replacement. The summary worksheet identifies loci where > 50% of the length was involved in a recombination event affecting the ancestor of at least one genome was inferred to have originated with *N. gonorrhoeae*. The “Dictionary” worksheet defines the columns used in the two “Recombinant loci” worksheets. (XLSX 662 kb)

## Abbreviations

CC11: ST-11 clonal complex
CDC: Centers for Disease Control and Prevention
CSF: Cerebrospinal fluid
GISP: Gonococcal Isolate Surveillance Project
GNID: gram-negative intracellular diplococci
MSM: Men who have sex with men
NAAT: Nucleic acid amplification testing
Nm: *Neisseria meningitidis*
NmC: *N. meningitidis* serogroup C
NmNG: *N. meningitidis* nongroupable
