# Supplementary figures and images for: Expansion of a urethritis-associated Neisseria meningitidis clade in the United States with concurrent acquisition of N. gonorrhoeae alleles
Source: BMC Genomics. 2018 Mar 2;19:176. doi: 10.1186/s12864-018-4560-x (PMC5834837; doi:10.1186/s12864-018-4560-x)

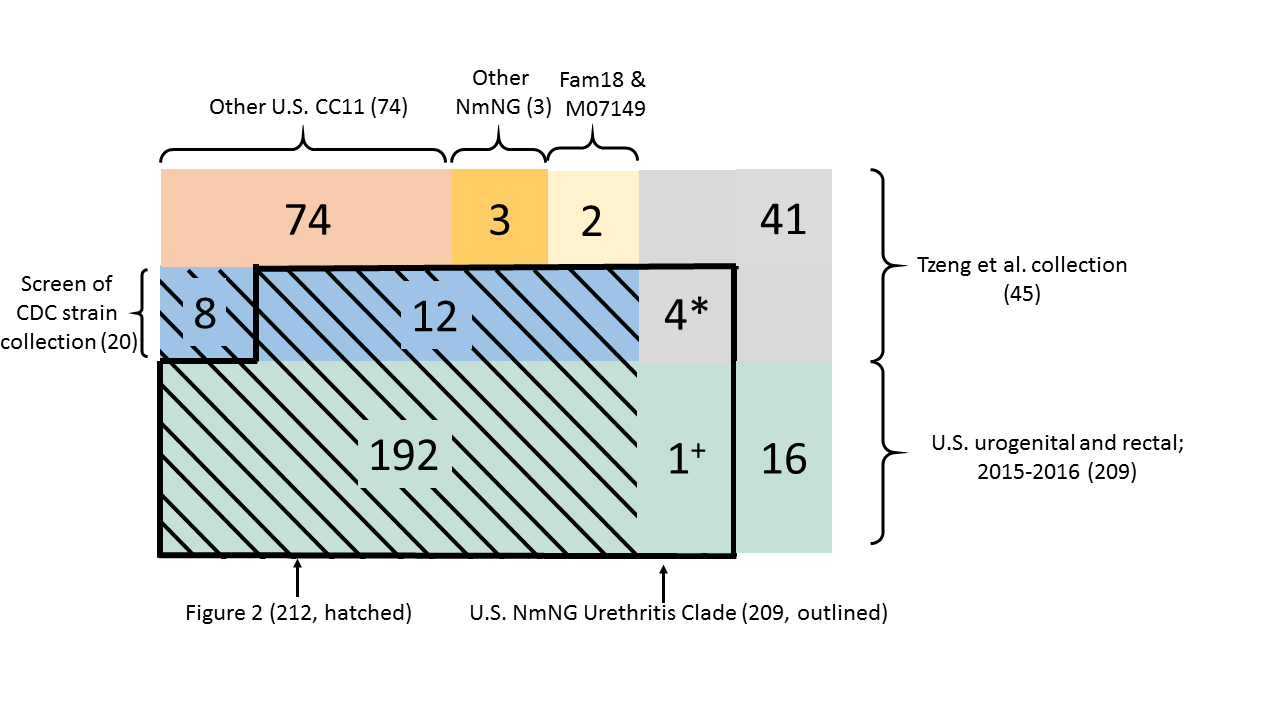

Supplement: Supplementary file 2 — Depiction of isolate collection included in each analysis. Regions are not to scale. All Isolates are included in phylogenetic analysis for Fig. 1 (n = 353). The bases for inclusion are listed in the margins. Isolates belonging to the U.S. NmNG urethritis clade are outlined, and isolates included in Fig. 2 are identified with diagonal hatching. Cross (+) identifies isolates that were excluded from analysis in Fig. 2 due to inappropriate data format. Asterisk (*) marks an isolate that was excluded from analysis in Fig. 2 due to insufficient data quality. (TIFF 127 kb) [file 12864_2018_4560_MOESM2_ESM.tif]
